# Atypical presentation of patients with chronic myeloid leukemia in chronic phase—Case report

**DOI:** 10.3389/fonc.2022.960914

**Published:** 2022-08-29

**Authors:** Florian Ramdohr, Alice Fabarius, Bettina Maier, Daniela Bretschneider, Anna Jauch, Astrid Monecke, Klaus H. Metzeler, Johannes W. G. Janssen, Richard F. Schlenk, Sabine Kayser

**Affiliations:** ^1^ Medical Clinic and Policlinic I, Hematology and Cellular Therapy, University Hospital Leipzig, Leipzig, Germany; ^2^ Department of Hematology and Oncology, University Hospital Mannheim, Heidelberg University, Mannheim, Germany; ^3^ Department of Internal Medicine V, University Hospital Heidelberg, Heidelberg, Germany; ^4^ Institute of Human Genetics, University Heidelberg, Heidelberg, Germany; ^5^ Department of Diagnostics, Institute of Pathology, University Hospital Leipzig, Leipzig, Germany; ^6^ NCT Trial Center, National Center of Tumor Diseases, German Cancer Research Center (DKFZ), Heidelberg, Germany; ^7^ Department of Medical Oncology, National Center of Tumor Diseases (NCT), Heidelberg University Hospital, Heidelberg, Germany

**Keywords:** chronic myedoid leukemia (CML), atypical transcripts, BCR-ABL +, e6a2, tyrosine kinase inhibitors, outcome

## Abstract

The presence of the translocation t(9;22)(q34;q11), leading to the *BCR::ABL1* fusion transcript, is the hallmark of chronic myeloid leukemia (CML). Nevertheless, atypical presentation at diagnosis can be challenging. However, although most patients with CML are diagnosed with the e13a2 or e14a2 *BCR::ABL1* fusion transcripts, about 5% of them carry rare *BCR::ABL1* fusion transcripts, such as e19a2, e8a2, e13a3, e14a3, e1a3, and e6a2. In particular, the e6a2 fusion transcript has been associated with clinically aggressive disease frequently presenting in accelerated or blast crisis phases. To date, there is limited evidence on the efficacy of front-line second-generation tyrosine kinase inhibitors for this genotype. Here, we report two patients, in whom the diagnosis of CML was challenging. The use of primers recognizing more distant exons from the common *BCR::ABL1* breakpoint region correctly identified the atypical *BCR::ABL1* e6a2 fusion transcript. Treatment with the second-generation tyrosine kinase inhibitor nilotinib was effective in our patient expressing the atypical e6a2 *BCR::ABL1* fusion transcript.

## Introduction

Chronic myeloid leukemia (CML) is a malignant disease of the hematologic stem cell. The hallmark of the disease is the translocation t(9;22)(q34.1;q11.2), leading to the *BCR::ABL1* fusion transcript (1–3). Nearly half of the patients are asymptomatic at diagnosis and discovered when a white blood cell (WBC) count performed as part of a routine analysis is found to be abnormal. Additionally, most patients are diagnosed within chronic phase and only roughly 5% are initially diagnosed with accelerated or blast phase.

Treatment with tyrosine kinase inhibitors (TKIs), particularly of the second generation, such as nilotinib or dasatinib, leads to high molecular remission rates (4).

However, there is growing evidence of cases with an atypical clinical presentation, mostly related to alternative transcripts, accounting for less than 1% of CML patients and their clinical significance is still under investigation (5–8). These uncommon variant transcripts can result in phenotypic variability and affect response to TKI therapy (9). The atypical e6a2 *BCR::ABL1* transcript produces a rare fusion protein, which confers a poor prognosis in CML due to its association with aggressive phenotype and early transformation, perhaps due to the lack of an important regulatory *BCR* sequence within the fusion proteins (6).

Atypical presentations of CML can further be due to an unusual laboratory presentation or a concurrent second malignant disease, obscuring the diagnosis and thus hampering a highly curable disease. We here present two cases of CML with atypical clinical presentation.

## Case description

### Case 1

**Figure 1:**
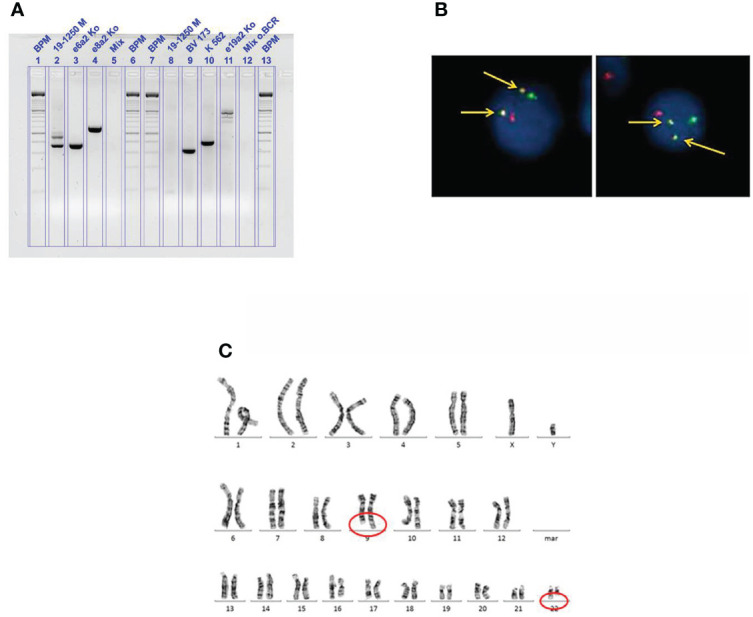
**(A)** Fluorescence *in situ* hybridization on bone marrow from case 1 showing a reciprocal *BCR::ABL1* fusion (arrows, indicated in yellow); red signal, probe for ABL on the long arm of chromosome 9 (9q34); green signal, probe for BCR on the long arm of chromosome 22 (22q11). **(B)** Cytogenetic analysis on bone marrow from case 1 showing the translocation t(9;22)(q34;q11). **(C)** Agarose gel analysis from cDNA extracted from peripheral blood from case 1 showing a positive result for the rare e6a2 transcript. BPM, base pair ladder (lines 1, 6, 7, and 13); 19-1250 M (lines 2 and 8), cDNA from patient 1; BV 173, K562, e19a2 (lines 9, 10, and 11), cDNA from *BCR::ABL1* positive cell-line controls; Mix (lines 5 and 12), negative controls; rare transcript controls (lines 3 and 4), e6a2 and e8a2.

The first patient, a 50-year-old man, presented in February 2019 due to fatigue. Laboratory evaluation revealed an elevated WBC count of 264.7 × 10^9^/L, anemia of 7.4 g/dl, and increased platelets of 1,376 × 10^9^/L. Differential blood cell count revealed a left shift with 10% myeloid blast cells, whereas basophils and eosinophils were absent. LDH value was heavily increased with 1,418 U/L (range, 125–248 U/L) and uric acid was 8.6 g/dl (range, 3.5–7.0 mg/dl). Bone marrow evaluation revealed 4% myeloid blast cells. Abdominal ultrasound showed hepatosplenomegaly with a spleen size of 18 × 9 cm as well as a liver size of 21 cm (craniocaudal). In suspicion of CML in chronic phase, therapy with hydroxyurea was started with 4 g/daily as well as supportive care to prevent tumor lysis syndrome. After transfusion of two packed red blood cells, the hemoglobin value rose sufficiently to 9.2 g/dl. Astonishingly, the initial PCR analysis, targeting the typical isoforms p210 and p190, was negative for *BCR::ABL*. However, fluorescence *in situ* hybridization (FISH) analysis on bone marrow revealed a reciprocal *BCR::ABL1* fusion in 99% of 500 cells counted (**Figure 1A**) and cytogenetic analysis was positive for translocation t(9;22)(q34;q11) in 14 metaphases as well (**Figure 1B**). Thus, the diagnosis of CML in chronic phase was made. The Sokal and ELTS scores were high risk. Interestingly, in a second attempt, quantitative real-time PCR, targeting the rare transcript e6a2, revealed a *BCR::ABL/ABL1* transcript ratio of 40.563% (**Figure 1C**). Unfortunately, patients with rare *BCR::ABL* transcripts cannot be measured according to International Scale (IS), since it is standardized only for those with standard transcripts e13a2 and e14a2. Of course, all analyses were performed according to good clinical and laboratory practice, e.g., all analyses were performed as double analyses, including negative and positive controls from peripheral blood of healthy persons (**Figure 1**). Scoring of molecular responses were calculated as published (10). After 9 days of hydroxyurea, WBC count declined sufficiently to 29/nl; however, the platelets rose from initially 1,376 × 10^9^/L to 1,950 × 10^9^/L. Hydroxyurea was stopped and switched to the second-generation TKI nilotinib 300 mg twice daily within the NILOdeepR trial (ClinicalTrials.gov Identifier: NCT02546674).

However, 10 days after TKI initiation, the platelets rose further to 3,262 × 10^9^/L, whereas WBC count remained faintly stable at 32 × 10^9^/L. After one more week of TKI therapy, platelets declined to 1,018 × 10^9^/L and finally to 456 × 10^9^/L after one more week of therapy. Consistently, WBC count dropped to 10 × 10^9^/L and finally normalized. Hemoglobin value slightly decreased to minimally 8.1 g/dl, but started to raise spontaneously after a few weeks to 10.7 g/dl. Bone marrow evaluation after 3 months of therapy revealed a complete cytogenetic remission (CCR) with normal cytogenetics in 25 metaphases and a decline of the *BCR::ABL/ABL1* transcript ratio to 0.063% by quantitative PCR analysis. Peripheral blood count had normalized as well and both spleen and liver were not palpable. After 6 months of TKI therapy, the patient was still in CCR and also in molecular remission with 0% *BCR::ABL/ABL1* transcripts by quantitative PCR analysis. After 1 year of treatment with nilotinib within the NILOdeepR trial (ClinicalTrials.gov Identifier: NCT02546674), the patient achieved deep molecular remission (MR4.5) and continues in deep remission (MR5) since December 2021 (last follow-up in March 2022).

The patient was recently interviewed on his view concerning the disease and treatment approach. He described himself as being positive-thinking and future-oriented. At the time of CML diagnosis, he was worried, but his familiar background (two daughters) stimulated him to fight. He felt relieved that the whole course of therapy could be conducted ambulatory. His optimism grew already after the first month, when the blood values declined as expected. He is in deep molecular remission without any symptoms. Currently, the patient is working again, feels healthy, and is confident about his future.

### Case 2

The second patient, a 79-year-old man, was initially diagnosed with a triple-negative (*JAK2, CALR, MPL*) primary myelofibrosis in November 2019. Initially, cytogenetic analysis was not successful. At diagnosis, WBC count was only slightly increased (10.5 × 10^9^/L; range, 3.5–9.8 × 10^9^/L), and hemoglobin value (11.6 g/dl; range, 13.5–17.5 g/dl) as well as platelet counts slightly decreased (130 × 10^9^/L; range, 140–360 × 10^9^/L). Apart from that, the differential blood cell count was normal. Colonoscopy and x-ray of the chest were unremarkable. Due to hyperchromic, macrocytic anemia, therapy with vitamin B12 1,000 µg was initiated subcutaneously every 4 weeks. Gastroscopy 3 months later showed *Helicobacter pylori*-negative type A gastritis. Flow cytometry revealed 4% myeloid blast cells within peripheral blood. At that time, the patient received a transfusion with two packed red blood cells for the first time. Abdominal ultrasound showed mild hepatosplenomegaly with a spleen size of 14.5 × 7 cm. Bone marrow evaluation was performed in November 2020 due to progressive WBC count, and anemia was suggestive of primary myelofibrosis. PCR analysis was negative for *BCR::ABL*, *JAK2*, *CALR*, and *MPL* mutations. Astonishingly, flow cytometry was unremarkable at that time. CT scan in December 2020 showed progressive splenomegaly with a spleen size of 17 cm. A therapy with erythropoietin 20,000 IE once weekly was initiated in February 2021 and vitamin B12 was stopped. The patient did not receive a JAK2 inhibitor. Due to a progressive WBC count of 44.2 × 10^9^/L, bone marrow evaluation was repeated in June 2021. Histopathology revealed a hypercellular bone marrow with pronounced megakaryo- and myelopoiesis, whereas erythropoiesis was strongly decreased, mild fibrosis (grade I), and 2% myeloid blast cells. Mast cells were not elevated in the bone marrow or peripheral blood. In molecular analysis, mutations in *ASXL1, EZH2, FLT3, RUNX1, STAG2, and TET2* could be detected. Thus, diagnosis was switched to atypical CML. Cytogenetic analysis revealed a trisomy 8 in 13 as well as a normal karyotype in 7 metaphases (47,XY+8[13]/46,XY[7]). Medical history included arterial hypertension, glaucoma, chronic renal failure (grade IV KDIGO), and benign prostate hyperplasia.

In August 2021, the patient was admitted to our department after syncope. At admission, his performance status was slightly decreased (ECOG 2). He showed signs of anemia with tachycardia (91 bpm at rest), pale skin, and a previously unknown cardiac murmur. Moreover, multiple purple lesions, particularly at the head, were visible, which developed within a few weeks before admission. Physical examination revealed palpable hepatosplenomegaly.

The reason for the syncope turned out to be anemia of 6.6 g/dl (range, 13.5–17.5 g/dl). Furthermore, laboratory evaluation showed massively elevated WBC count of 249.4 × 10^9^/L (range, 3.5–9.8 × 10^9^/L), mild decreased platelets (127 × 10^9^/L; range, 140–360 × 10^9^/L), and an elevated lactate dehydrogenase (LDH) value of 1101.6 U/L (range, 125–248 U/L). Differential blood cell count showed a neutrophil left shift including 4% myeloid blast cells and a basophile count of 1.5%.

Due to leukocytosis, we started a cytoreductive treatment with hydroxycarbamide. With a daily dosage of 5 g, WBC count decreased from 249.4 to 97.6 × 10^9^/L within 1 week. To prevent tumor lysis syndrome and due to known renal impairment, intravenous fluids, furosemide, and allopurinol as co-medications were administered.

Bone marrow evaluation showed hypoplastic erythropoiesis as well as myeloid blast cells of 10%, consistent with the previous diagnosis of atypical CML. However, cytogenetic analysis now revealed a balanced translocation t(9;22)(q34;q11) in 21 of 34 metaphases as well as a second, Philadelphia chromosome-negative clone with trisomy 8 in 3 and a normal karyotype in 10 metaphases (46,XY,t(9;22)(q34;q11)[21]/47,XY,+8[3]/46,XY[10]). Molecularly, *BCR::ABL* positivity was confirmed by PCR and FISH analyses (FISH positive in 68% of the interphase cells). The Sokal and ELTS scores were high risk.

To evaluate the skin lesions, we performed a skin biopsy. Histopathology showed a CD33-negative, CD34-negative, and CD117-positive myeloid blast cell population, which we interpreted within the context of the underlying CML.

Due to palpable hepatosplenomegaly, we performed an abdominal ultrasound, which confirmed the finding and additionally showed two splenic infarctions.

In summary, the diagnosis of *BCR::ABL-*positive CML in chronic phase was made and treatment with imatinib was started and hydroxycarbamide was terminated, once a WBC count of lower than 40 × 10^9^/L was reached. In the following days, the performance status increased (ECOG 1) and the patient was discharged with a follow-up appointment with his local hemato-oncologist.

Re-admission to our hospital followed 18 days later with a drastically decreased performance status (ECOG 3) and acute on chronic renal failure. Although imatinib had been taken daily, WBC count had increased again to 47.7 × 10^9^/L as compared to 23 × 10^9^/L at discharge. Platelets were within normal range, but the patient was still anemic (hemoglobin value of 7.3 g/dl). Additionally, there was a mixed response of the skin lesions: some had disappeared, others increased, and some new lesions had occurred.

To exclude a treatment failure, we performed bone marrow evaluation, which again showed CML in chronic phase. No resistance mutation could be detected by PCR analysis, and the *BCR::ABL*-negative trisomy 8 subclone was found in 48% of the interphase cells by FISH analysis and in 1 of 25 metaphases (46,XY,t(9;22)(q34;q11)[9]/47,XY,+8[1]/46,XY[15]). We continued treatment with imatinib and re-initiated hydroxycarbamide due to the increased WBC count.

After a few days, the patient developed pneumocystis jirovecii pneumonia, which was treated with atovaquone/proguanil hydrochloride as well as corticosteroids. Due to progressive respiratory insufficiency, the patient had to be transferred to the intensive care unit. Unfortunately, the patient developed multi-organ with leading pulmonary failure. Pleural effusions were drained and showed myeloid blast cells. The patient had to be intubated and noradrenaline had to be used for circulatory support. Unfortunately, the patient died several days later due to multi-organ failure. An autopsy was performed, confirming pneumocystis jirovecii pneumonia (*via* positive PCR), but also showed several other interesting aspects.

**Figure 2:**
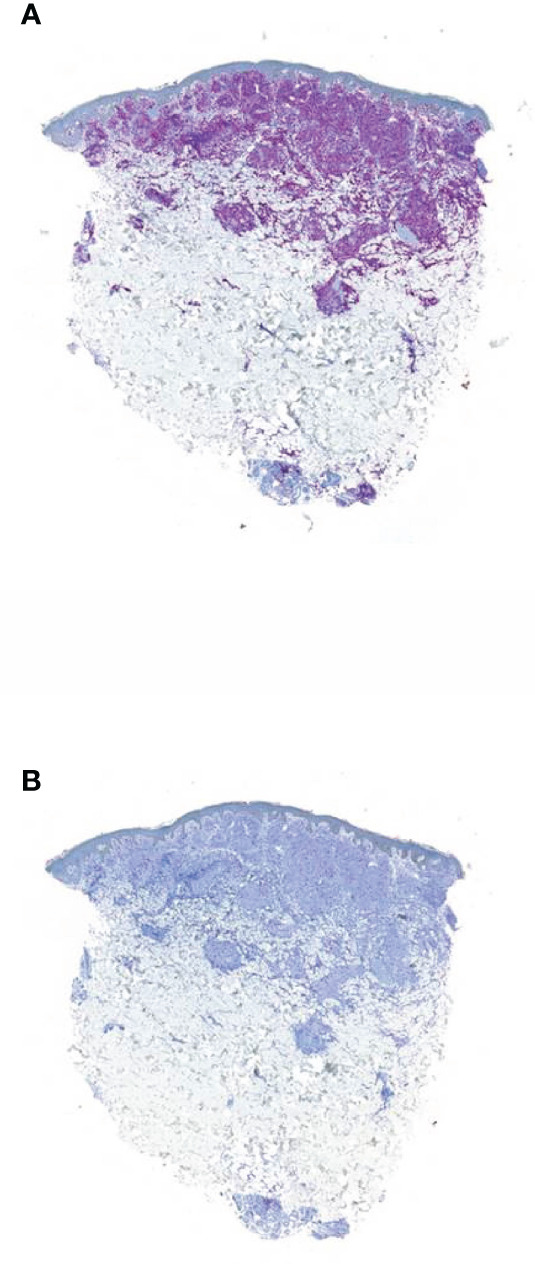
Immunohistochemistry of the skin biopsy from the second patient. **(A)** Infiltration of small cellular lymphoid cell components in CD 8 staining; scale bar, 500 µm. **(B)** Antigen loss of CD2 of the lymphoid infiltrates detected in hematoxylin and eosin staining; scale bar, 500 µm.

First, the skin effusions showed a CD5, CD7, and CD8 expression (**Figure 2A**), leading to a CD8+ T-cell lymphoma of the skin with aberrant expression of CD117 and loss of CD2 (**Figure 2B**). Initially, CD117 positivity led to the assumption of CML skin involvement. The T-cell lymphoma was mainly limited to the skin, explaining the progressive skin nodules during treatment with imatinib.

**Figure 3:**
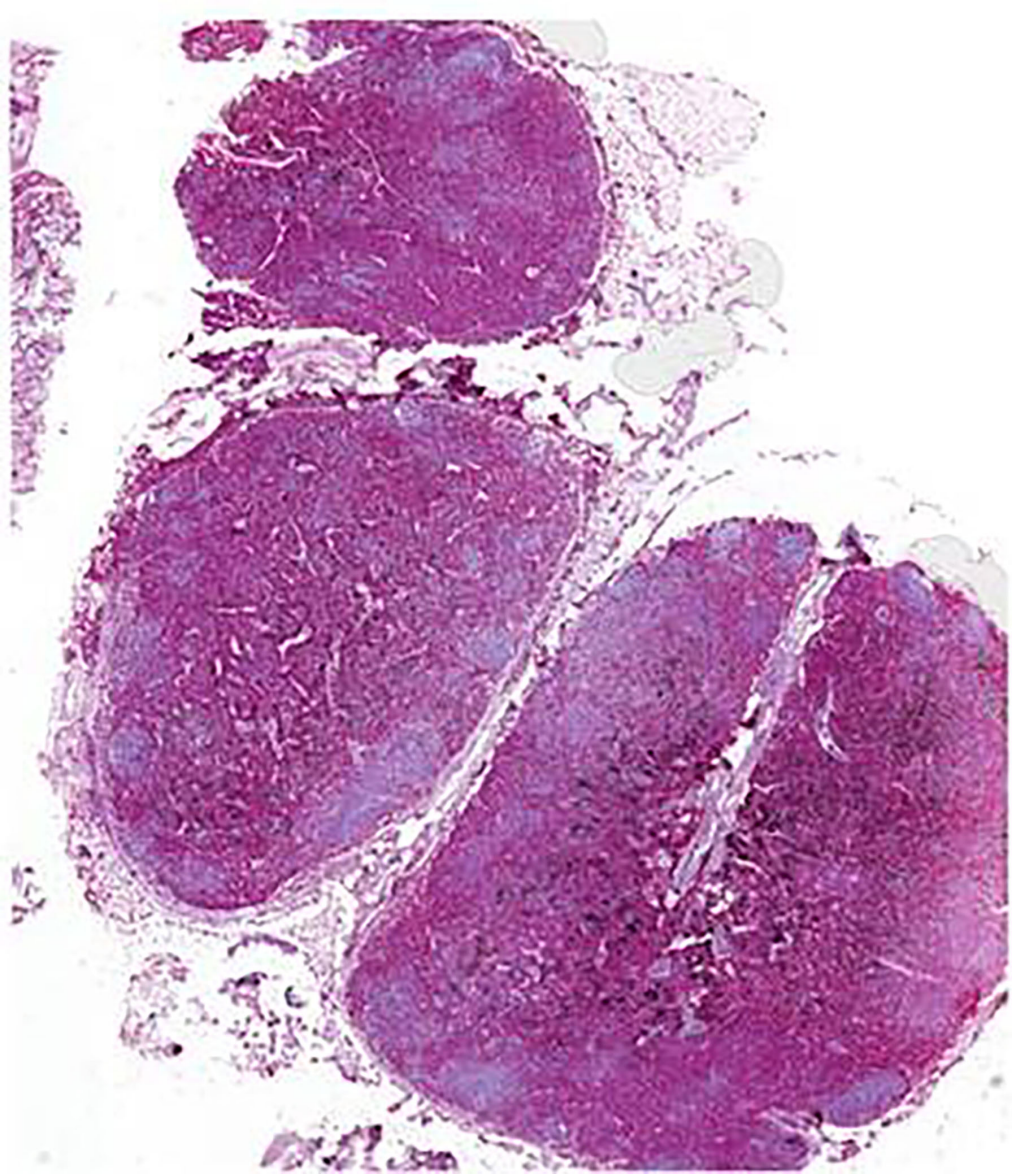
Lymph node biopsy from case 2 with myeloperoxidase staining showing infiltration of myeloid precursors; scale bar, 1,000 µm.

**Figure 4:**
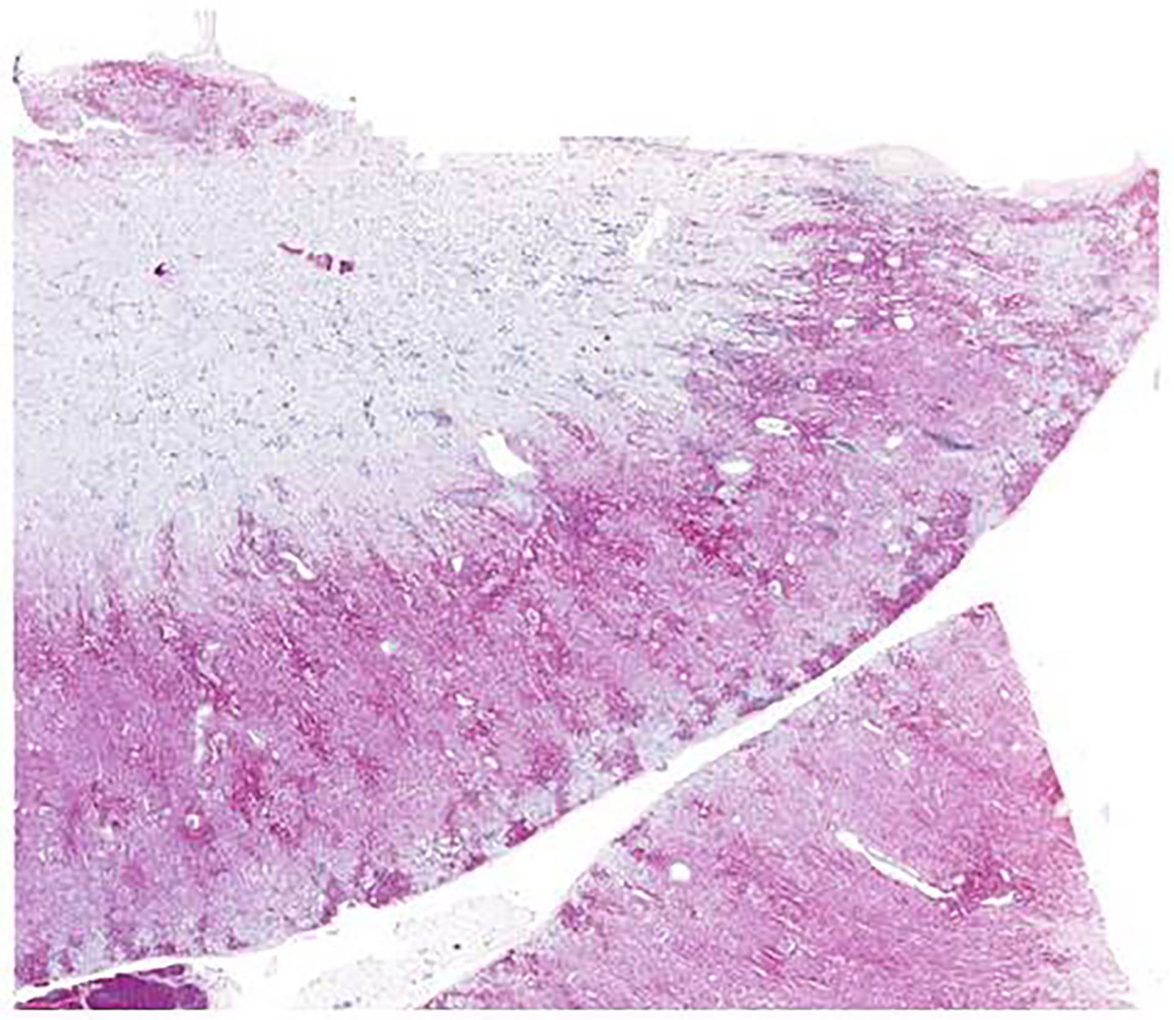
Renal biopsy from case 2 with myeloperoxidase staining showing infiltration of myeloid precursors; scale bar, 2,000 µm.

Second, multiple organs showed a severe infiltration with myeloid precursors (**Figure 3**). Lung involvement (about 50% of the parenchyma), splenic involvement (90%), hepatic involvement (20%), and renal involvement (30%) (**Figure 4**) possibly explain the relatively sudden worsening of the performance status and multi-organ failure. Moreover, multiple lymph nodes, pleura, thyroid, prostate, and testicles were involved as well. However, the aggressive course of the disease with the invasion of multiple organs remains an obstacle.

Retrospective mutational analysis by next-generation sequencing at progressive disease showed mutations in *ASXL1* (c.1720-1G>C), *EZH2* (c.2069G>A; c.132delC), *STAG2* (c.436C>T; c2353G>T), and *TET2* (c.5038C>T). Additionally, two different *NRAS* mutations (c.35G>T; c.35G>A) with allele frequencies of 3% and 12% could be detected, whereas the mutations in *FLT3* and *RUNX1*, present at initial diagnosis, could not be detected.

## Discussion

Our cases highlight the importance of a thorough investigation of the underlying diagnosis in patients with clinically unclear hematologic diseases but suspected CML.

In fact, the use of conventional multiplex RT-PCR usually fails to detect uncommon *BCR::ABL1* rearrangements due to the generation of atypical PCR products, which are often interpreted as nonspecific and may lead to misdiagnosis, thus excluding the patient from targeted therapy. While atypical transcripts in CML patients are increasingly reported (11–14), the outcome seems to be adverse in some of the more “frequent rare” mutations such as e1a2 (15, 16).

In 2019, Xue et al. evaluated the incidence and prognostic significance of CML patients with rare transcripts in a large cohort of 2,331 patients (15). Rare transcripts were found in 40 (1.7%) patients, including e1a2 (0.9%), e19a2 (0.4%), e13a3 (0.1%), and e14a3 (0.3%). Compared to patients with the typical transcript, those with the e1a2 transcript had an inferior response to TKIs [complete cytogenetic response rate (CCyR), 19.0% vs. 79.9%; *p* < 0.001] and an inferior 3-year overall survival (OS) rate (83.9% vs. 98.7%; *p* < 0.001). Patients with the e19a2 transcript had a high rate of early optimal response to TKIs, but most of them later lost CCyR due to *BCR::ABL1* mutations, resulting in a poor prognosis. Patients with the e13a3 or e14a3 transcripts responded well to TKIs and had a good outcome. However, the low patient number needs to be taken into account, when interpreting the data. Similarly, Gong et al. reported an inferior outcome for patients with e1a2 transcripts as compared to those with typical transcripts, such as e13a2 and e14a2 (16). Patients with e1a2 were more likely to develop CML with blast phase (61% vs 17.6%; *p* < 0.001) and the transcript was predictive for inferior OS (*p* = 0.002) as compared to patients with typical transcripts (16). In other cases with rare transcripts, the impact on outcome remains unclear due to their rarity and lack of data.

To date, there are only limited data on outcome of CML patients with the *BCR::ABL1* e6a2 transcript. Several case reports suggest an aggressive course of the disease with resistance to imatinib (17, 18) and dasatinib (18) treatment. In addition, three (50%) of six imatinib-treated CML patients with the *BCR::ABL* e6a2 transcript relapsed or did not respond (6, 19, 20). Two of them additionally developed imatinib resistance (19, 20) and one developed extramedullary disease (20). The short duration of response and rapid progression into advanced phase may indicate that the e6a2 *BCR::ABL* transcript has a higher transforming capacity than the standard major *BCR* transcript (e13a2 or e14a2 *BCR::ABL*). In our case, treatment with nilotinib within the NILOdeepR trial (ClinicalTrials.gov Identifier: NCT02546674), as second-generation TKI, led to a deep and enduring molecular remission, in line with a previous case report (21). Thus, the form of transcript should be considered when deciding on the optimal treatment approach. Our second case shows the diagnostic difficulty of an uncommon CML presentation. The initial diagnosis was a triple-negative primary myelofibrosis. The *JAK2* V617F mutation is specific for *BCR::ABL1* negative myeloproliferative neoplasm and occurs in approximately 50% of patients with primary myelofibrosis. However, mutational analysis was negative for *JAK2, CALR*, and *MPL* at diagnosis.

Initially, cytogenetic analyses revealed only trisomy 8 and no Philadelphia chromosome could be detected. However, repeated analysis in August 2021 showed the balanced translocation t(9;22) in 21 metaphases and in a small subclone trisomy 8. Thus, primary myelofibrosis and CML with *BCR::ABL* were separate clones, which evolved from a mutual precursor cell. This precursor was characterized by mutations in *TET2, EZH2, ASXL1*, and *STAG2*. Repeated analyses by next-generation sequencing revealed some overlapping mutations, but also discrepancies, since *NRAS* mutations evolved, whereas mutations in *FLT3* and *RUNX1*, present at initial diagnosis, could not be detected. In summary, this is a case of “branching” rather than “linear” evolution.

In addition, the secondary neoplasia of a T-cell non-Hodgkin’s lymphoma (T-NHL) obscured the diagnosis, leading to the assumption of initial treatment failure. Although concurrent neoplasias are described in CML patients (22–25), they are still a diagnostic as well as therapeutic challenge. In our case, the atypical phenotype led to the assumption of CML infiltration of the skin, whereas autopsy revealed the diagnosis of a CD2-negative T-NHL. Interestingly, in autopsy, most organs showed an abundant infiltration with myeloid precursors, while sparing the skin, whereas the T-cell lymphoma mainly infiltrated the skin. The reason for this organ tropism remains elusive.

One reason for the large-scale infiltration of CML, despite being in chronic phase, might be driven by molecular abnormalities, such as *ASXL1* or *RUNX1* (26, 27). The impact on outcome of the other described mutations remains unclear (28–30). The known resistance to imatinib is consistent with our case and underlines the importance to evaluate the molecular profile, particularly in “atypical typical” cases.

In conclusion, in patients with suspected CML, the presence of rare transcripts needs to be taken into account to prevent the possibility of a misdiagnosis, thus preventing the patient from targeted therapy. Treatment with second-generation TKIs, such as nilotinib, seems to be effective in patients with the rare transcript e6a2. However, larger data, ideally from multicenter clinical trials, are needed in order to define the best treatment choice for patients with these rare transcripts.

## Data availability statement

The raw data supporting the conclusions of this article will be made available upon reasonable request.

## Ethics statement

Ethical review and approval was not required for the study on human participants in accordance with the local legislation and institutional requirements. Patients declare on their admission that their data may be used in anonymised form for scientific evaluations and for publications.

## Author contributions

FR and SK were responsible for the concept of this paper, contributed to the literature search data collection, treated the patients, analysed and interpreted data, and wrote the manuscript. RS treated the patient and critically revised the manuscript. AF, BM, DB, AJ, AM, KM, and JWGJ performed research and critically revised the manuscript. All authors approved the submission.

## Funding

The authors acknowledge support from the University of Leipzig within the program of Open Access Publishing.

## Conflict of interest

The authors declare that the research was conducted in the absence of any commercial or financial relationships that could be construed as a potential conflict of interest.

## Publisher’s note

All claims expressed in this article are solely those of the authors and do not necessarily represent those of their affiliated organizations, or those of the publisher, the editors and the reviewers. Any product that may be evaluated in this article, or claim that may be made by its manufacturer, is not guaranteed or endorsed by the publisher.
